# A New Approach for Evaluation of Cardiovascular Fitness and Cardiac Responses to Maximal Exercise Test in Master Runners: A Cross-Sectional Study

**DOI:** 10.3390/jcm11061648

**Published:** 2022-03-16

**Authors:** Pedro Á. Latorre-Román, Felipe García-Pinillos, Jesús Salas Sánchez, Marcos Muñoz Jiménez, Víctor Serrano Huete, Melchor Martínez Redondo, Jerónimo Aragón Vela, Juan A. Párraga-Montilla

**Affiliations:** 1 Department of Corporal Expression, University of Jaen, 23071 Jaen, Spain; platorre@ujaen.es (P.Á.L.-R.); mmjimene@ujaen.es (M.M.J.); jparraga@ujaen.es (J.A.P.-M.); 2 Department of Physical Education, Sports and Recreation, Universidad de La Frontera, Temuco 4780000, Chile; fegarpi@gmail.com; 3 Department of Physical Education and Sports, University of Granada, 18071 Granada, Spain; 4 Universidad Autónoma de Chile, Santiago 7500912, Chile; jsalas@ujaen.es; 5 Faculty of Humanities and Social Sciences, International University Isabel I, 09003 Burgos, Spain; victor.serrano@ui1.es; 6 Consejería de Educación de Andalucía, CEIP Doctor Fleming, 23500 Jodar, Spain; melchor_mr@hotmail.com; 7 Department Physiology, Faculty of Sport Sciences, University of Granada, 18071 Granada, Spain; 8 Department of Nutrition, Exercise and Sports (NEXS), University of Copenhagen, 1165 Copenhagen, Denmark

**Keywords:** heart rate, master runners, exercise, physical activity, active life expectancy

## Abstract

The aim of this study was to analyze the cardiac autonomic function at rest, at maximum exercise, and in recovery after exercise and to determine sex-specific and age-specific values for resting heart rate (RHR), hear rate (HR)-peak, HR recovery (HRR), and HR variability at rest in master runners. Fifty endurance runners (21 women) participated in this study (43.28 ± 5.25 years). The subjects came from different athletic clubs in Andalusia (Spain), and the testing protocol was performed in-season. A 3-km running test was performed and the cardiovascular response was monitored. Regarding sex, no significant differences were found regarding cardiovascular autonomic function at rest, during exercise, and following maximal exercise, only at rest, the standard deviation of all R-R intervals and low frequency values displayed significantly (*p* < 0.05) lower scores in women. 46% of athletes showed an RHR < 60 bpm. Additionally, HR-peak showed a significant correlation with age (r = −0.369; *p* = 0.009) and HRR_5min_ (r = 0.476, *p* = 0.001). Also, endurance performance was inversely associated with obesity traits and cardiometabolic risk factors. In summary, age, sex, fitness, or anthropometrics characteristics did not show a relevant influence on cardiovascular autonomic modulation in master runners. However, the 3-km performance displayed a significant negative association with several factors of cardiometabolic risk.

## 1. Introduction

Life expectancy in developed countries has been increased in recent decades [1]. This rising life expectancy is due to improvements in factors such as nutrition and development in modern medicine, but especially an increase in the practice of physical activity (PA) [2]. The presence of age-associated health risks demands exploration on how to encourage successful aging [3]. Specifically, athletes have been proposed as an ideal model of aging due to their involvement in high-intensity activities [4,5]. Master athletes, men and women older than 35 years who participate in competitive sports, form a rapidly growing population that is increasingly encountered in clinical cardiovascular practice [6]. These athletes represent the human capacity to maintain certain physical performance and physiological functions throughout the years [7]. Through high training volumes and intensities, athletes maintain high PA levels and show healthier results than age-matched non-athletes [8]. In particular, even running <51 min, <9.65 km, 1 to 2 times, or <9.65 m/h per week has been shown to reduce the risk of mortality, related with not running; in addition, persistent runners display the most significant benefits, with 29% and 50% lower risks of all-cause and cardiovascular mortality, respectively, compared with non-runners [9]. Therefore, the master athlete exemplifies a remarkable model for studying unadulterated aging, without the contaminant effects of a sedentary lifestyle and the lack of fitness that accentuate the aging process [10].

Aging is associated with cardiac autonomic function impairment due to excessive sympathetic activation and/or a reduced vagal outflow, which is related to several cardiovascular diseases [11]. In addition, aging reduces the capacity for physical exertion, nevertheless, it is not clear to what extent this can be attributed to a decrease in cardiac reserve [12,13]. The effect of aging on autonomic nervous system cardiac control is progressive and continuous during an 18–80 years’ age range [13]. In this regard, alterations in cardiac function which exceed the identified limits for aging changes for healthy older people are possibly an expression of the interaction of physical deconditioning and cardiovascular disease [12]. In particular, variables such as heart rate recovery (HRR) and heart rate variability (HRV) responses to exercise are known to be negatively influenced by age [14].

The long-term response of heart rate (HR) to training elicits positive changes in chronotropic function, as well as a decrease in resting heart rate (RHR) in submaximal HR and an increase in HRR [15]. Accordingly, there is strong evidence showing that RHR, HR-peak, HRR, and HRV may be considered an index of cardiorespiratory fitness (CRF) and are useful tools for the assessment of cardiac autonomic function as a biomarker of cardiovascular health [16,17]. Furthermore, the analysis of HRV values in athletes who are free of comorbid diseases may help to distinguish age-associated changes in HRV that are due to decreased fitness and obesity, versus those that are due to primary aging per se [18].

In this regard, the measure of the acute cardiovascular response should be considered in adult runners during endurance tests in terms of the early identification of high-risk subjects due to possible abnormalities in the control of HR. So far, limited studies have analyzed the exercise HR profile in master athletes [17,19]. Likewise, a limited number of studies have provided relevant reference values for short-term HRV [11] or have analyzed sex differences [19,20,21]. Therefore, advances in the description of the physiological and cardiovascular characteristics of master athletes are necessary for terms of prevention of adverse events, and performance improvement, as well as to determine the positive or negative effects of regular training on the health of the runner.

Considering the positive effects of PA on ageing, authors hypothesize that amateur running programs (4–5 times/week) in master runners would induce a healthy cardiac autonomic state, without distinction of sex. This information would be of great help in the clinical field, such as this reference values could be used as a ‘warning signal’, with development of individualized health programs based on measurable values of HR. Consequently, the main purpose of this study was to analyze cardiac autonomic function at rest, at maximum exercise, and during the recovery phase in healthy Caucasian master runners. The second aim was to determine the association of HR parameters with cardiorespiratory fitness and anthropometric variables.

## 2. Materials and Methods

### 2.1. Experimental Approach to the Problem

This study was designed to examine the autonomic cardiovascular modulation at rest, during the maximal exercise test, and recovery after exercise in well-trained master runners according to age and sex. Fifty trained endurance runners performed a 3-km time trial. Performance was registered and used for the subsequent analysis. Finally, in order to test whether there is any relationship between the different variables used several correlations were made between autonomic cardiovascular modulations, cardiorespiratory performance, and cardiometabolic risk.

### 2.2. Participants

Fifty trained endurance runners (Men, *n* = 29: age = 43.86 ± 5.35 years; Women, *n* = 21: age = 42.47 ± 5.26 years) voluntarily participated in this study. The subjects came from different athletic clubs in Andalusia (Spain), and the assessment protocol was performed in-season. The participants of this study had performed a mean of 7.18 ± 6.24 years of training, with a program of 4.09 ± 1.05 weekly training sessions and an average distance of 50.06 ± 1.05 km. Inclusion criteria were as follows: (i) all subjects were healthy and none were taking any medication, (ii) the subjects had participated regularly in aerobic training at least four times per week during the last 2 years and were completing weekly mileage above 40 km, and (iii) the subjects had no history of injury in the previous six months that would limit training. About exclusion criteria, subjects with cardiorespiratory pathologies that affect cardiovascular performance, such as asthma, allergies, diabetes, or other cardiac pathologies, were not included. In addition, according to Cole et al. [22], resting systolic blood pressure less than 90 or greater than 200 mm Hg and resting diastolic blood pressure greater than 120 mm Hg were other exclusion criteria. After receiving detailed information on the objectives and procedure of the study, each participant signed an informed consent form for participation, which complied with the ethical standards of the World Medical Association Declaration of Helsinki (2013). The study was approved by the local Ethics Committee of the University of Granada (nº: 2288/CEIH/2021).

### 2.3. Material and Testing

#### 2.3.1. Anthropometric Variables

Body mass (kg) was measured using a weighing scale (Seca 899, Hamburg, Germany), and body height (cm) was measured with a stadiometer (Seca 222, Hamburg, Germany). The body mass index (BMI) was calculated by dividing body mass (kg) by body height^2^ (in meters). Waist circumference (WC) was measured at the umbilical location by using non-elastic Ergonomic Circumference Measuring Tape (Seca 201, Germany; range 0–150 cm; accuracy: 1 mm). The waist-to-height ratio (WtHR) as a measure of cardiometabolic risk factors was obtained by dividing the WC (cm) by body height (cm) and was used as a tool to estimate the accumulation of fat in the central zone of the body. For the WtHR, the 0.5 cut-off point represented the best balance between sensitivity and specificity, indicating a WtHR greater than or equal to this value related to a higher cardiovascular risk [23]. Body fat was evaluated by TANITA 330 body composition analyzer (Tanita Corp., Tokyo, Japan). The BMI was categorized according to World Health Organization (WHO) criteria (<18.5 kg/m^2^, underweight; 18.5–24.9 kg/m^2^, normal-weight; 25.0–29.9 kg/m^2^, overweight; and ≥30 kg/m^2^, obese) [24].

#### 2.3.2. Cardiorespiratory Fitness

To determine maximal endurance performance, the subjects performed a 3-km running test on an outdoor 400-m track (field test). The time of the 3-km run was recorded. This test has been used in previous studies [25,26] to analyze endurance performance. Also, to obtain more information about the perceived exertion after completion of the 3-km test, the rate of perceived exertion (RPE) was recorded on a scale from 6 to 20 (with 6 being the lowest and 20 the highest intensity) immediately post-test [27].

#### 2.3.3. HR and HRV Measures

Regarding HR monitoring, a Firstbeat Bodyguard 2 of Firstbeat Technologies Ltd. (Jyväskylä, Finland) was used to record interbeat (RR) intervals. This monitor is a beat-to-beat HR recorder, which comprises a one-channel electrocardiogram obtained by two electrodes located on the chest. The monitor records at a sample rate of 1000 Hz and it has been validated with standard electrocardiogram equipment [28,29]. With the data obtained by the RR Interval Recorder, the study calculated HRV parameters through the algorithms included in the Software Firstbeat Sports of Firstbeat Technologies Ltd. (Jyväskylä, Finland).

HRV analysis included several algorithms, all of which are based on either the frequency or time domain. The frequency domain of HRV includes the determination of the high-frequency (HF) and low-frequency (LF) spectrum and providing the possibility of making an LF/HF ratio. HF may reflect parasympathetic activity, LF may indicate a combination of sympathetic and parasympathetic input and LF/HF may reflect sympathovagal balance [30]. Corresponding with Bobkowski et al. [31], we deliberately omitted the HF and LF in normalized units since there is a direct mathematical relationship between the two. On the other hand, the time domain analysis includes statistical measures that reflect parasympathetic activity such as the root-mean-square differences of successive heartbeat intervals (RMSSD) and the mean of the standard deviation of the NN interval (SDNN) [32,33]. The study performed RR interval data collection following the recommendations of the European Society of Cardiology and the North American Society of Pacing and Electrophysiology [33] for HRV analysis. In addition, the RR interval data and the previously mentioned software were also applied for the calculation of RHR HR-Peak, HRR, and HR Reserve. Moreover, chronotropic index (CI) was defined as an index of maximal predicted HR reserve (HRr) achieved; HRr is the difference between maximum age-predicted HR and HR at rest. In particular, the CI was accordingly defined as CI = (HR at peak exercise − resting HR)/[(220 − age) − resting HR)] [34], and chronotropic incompetence was said to be present when values of the chronotropic index were <0.80 [34]. HR was also recorded 1 and 5 min after the cessation of exercise, and HRR was calculated as the difference between HR-peak and HR at these recovery points. HRR_1min_ and HRR_5min_ represent the fast and slow phases of recovery time, respectively [35]. Based on previous work, we used HR references at rest, during exercise, and in the recovery time related to an increase in the risk of death [36,37]. Particularly displayed in people with a RHR that was more than 75 beats per minute (bpm); subjects with an increase in HR during exercise that was less than 89 bpm; and in subjects with a HRR_1min_ and HRR_5min_ of less than 25 beats and 75 beats per minute after the termination of exercise respectively [36,37].

#### 2.3.4. Procedures

Three days before the experiment, the participants received dietary recommendations from a nutritionist about the type of food they are allowed to eat. In this way, we avoid the consumption of products that could alter the data, such as caffeine. Participants were cited and tested on an outdoor 400-m track, individually, on one specific day. Testing was integrated into weekly training schedules, although the participants were advised to avoid strenuous exercise 72 h before. Before exercise testing, anthropometric variables were registered and RHR was measured after 10 min in a seated position. To stabilize the HR, runners were instructed to abstain from speaking or moving during the examination. The recording and analysis of resting HR and HRV at 0–10 min of rest in a seated position with spontaneous breathing were used [38]. Short recordings of HRV (5 min) in a non-laboratory setting are stable over months and therefore characteristic of an individual [39]. Participants were encouraged to stay as relaxed as possible during this procedure. Next, the subjects performed a standardized warm up, which consisted of 5–10 min of low-intensity running and five minutes of general mobility exercises and incremental runs. Five minutes after warming-up, the participants completed a 3-km time trial. Some instructions were given to participants (i.e., to cover the 3-km distance as fast as possible). The participants were motivated and encouraged to reach the best score possible in the test. During exercise, the HR-peak measurement was obtained in a subsample of 48 runners (96% of the whole group); participants were excluded if the HR monitor lost its signal during the 3-km test. The HR was also recorded at the first (HR_1min_) and fifth (HR_5min_) minute after the cessation of exercise, and HRR was calculated as the difference between HR-Peak and HR at these recovery points (HRR_1min_ and HRR_5min_). At the end of the 3-km test, all subjects immediately sat passively on a chair placed adjacent to the sports court. The time duration between the end of exercise and sitting was less than 10 s. Therefore, both pre-exercise and post-exercise measures of HR were performed in the seated position for at least 10 min (subjects were instructed to sit still, breath normally, and not engage in conversation). At the end of the exercise, the participants indicated their rate of perceived exertion (RPE) on a 6–20 scale [27]. The 3-km time trial performance was registered (in terms of time) and used for the subsequent analysis.

### 2.4. Statistical Analysis

Data were analyzed using SPSS, v.22.0 for Windows (SPSS Inc., Chicago, IL, USA). The significance level was set at *p* ≤ 0.05. Descriptive data are reported in terms of the mean and standard deviation (SD), and percentage (%). Normality was tested using the Kolmogorov-Smirnov tests. The results of the normality test revealed that data required nonparametric tests for analysis both before and after log transformation. Differences in the anthropometric characteristics and CRF between men and women were analyzed using t-tests. Differences in the HR parameters between men and women were analyzed using the Mann–Whitney U-test. Qualitative variables are shown as proportions, compared using the Chi^2^ test. In addition, to verify the relationship between HR parameters with CRF and anthropometric variables, partial correlation analysis (adjusted by age and sex) were used. The magnitude of correlation between measurement variables was designated as: <0.1 (trivial), 0.1–0.3 (small), 0.3–0.5 (moderate), 0.5–0.7 (large), 0.7–0.9 (very large), and 0.9–1.0 (almost perfect) [40].

## 3. Results

**Figure 1:**
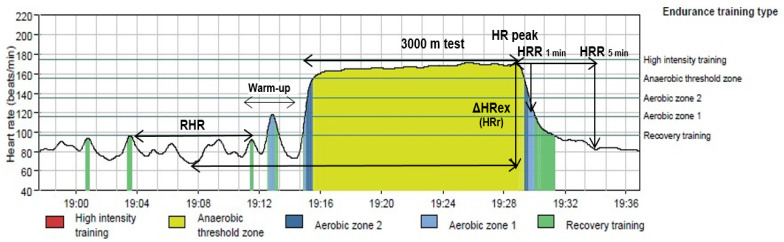
Heart rate (HR) profile at rest, during exercise and during recovery time. RHR: resting heart rate; HRR: heart rate recovery; ΔHRex: Increased heart rate during exercise.

Table 1 shows age, the anthropometric characteristic of participants and 3-km performance according to sex. No between-sex differences were found in age. Women exhibit higher values of body fat than men; however, men show higher BMI than women. According to the WHO classification of weight status, the overweight prevalence was 12% of participants. Regarding the 3-km time trial, men obtained the best performance. Figure 1 shows an example of HR profile at rest, during exercise and recovery times.

**Figure 2:**
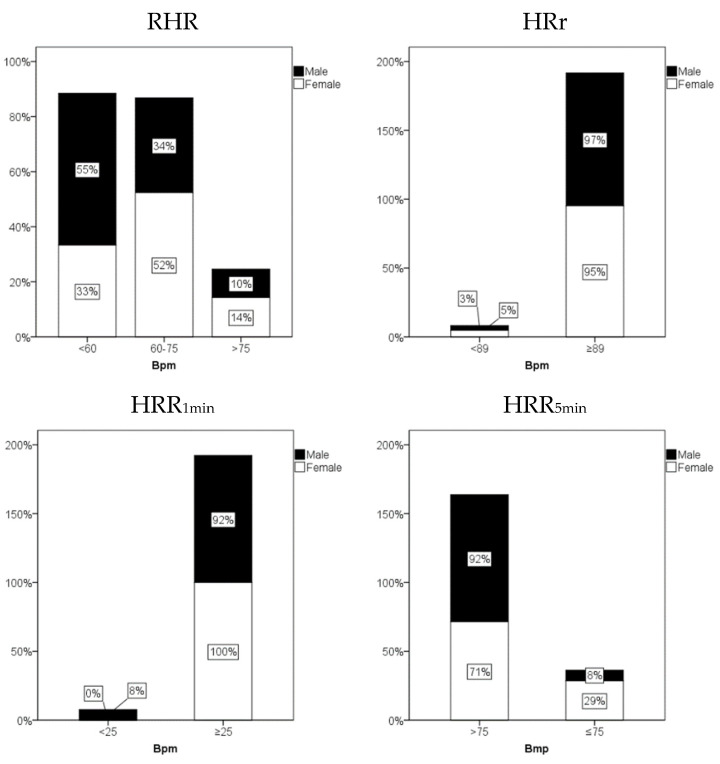
Percentage of athletes within each heart rate (HR) references (at rest, HR reserve and at recovery time) related to a higher risk of death. RHR, resting HR; HRR, HR recovery; HRr, heart rate reserve; Bpm, beats per minute.

Moreover, no significant differences were found regarding cardiovascular autonomic function at rest, during exercise, and following maximal exercise; only at rest did SDNN and LF values displayed significantly (*p* < 0.05) lower scores in women (Table 2). Figure 2 shows the percentage of athletes within each HR references values (at rest, during maximal exercise, and at recovery time) related to a higher risk of death. In all cardiovascular risk parameters, a low percentage of athletes present HR values compatible with a high risk of death. No significant differences were found between sexes in any HR variable. Controlling for age and sex, it can be seen that there is a very large correlation between RMSSD and RHR, and moderate/large correlation between HRR_5min_ and HRr; SDNN and RHR; HF and RHR and HRr and RMSSD (Table 3).

## 4. Discussion

The main purpose of this study was to analyze cardiac autonomic function at rest, at maximum exercise, and during the recovery phase in healthy Caucasian runners. The second aim was to determine the association of HR parameters with cardiorespiratory fitness and anthropometric variables. The major findings of this study were as follows. (i) No significant differences were found between sexes in the cardiac autonomic control during exercise and at recovery after exercise. However, at rest, women displayed lower values of SDNN and LF than men. (ii) A moderate significant correlation was found between HR-Peak and age. (iii) WtHR, BMI, and body fat did not influence cardiac response, although endurance performance was inversely associated with obesity traits and related cardiometabolic risk factors. (iv) Most runners have healthy cardiovascular autonomic modulation. Furthermore, we will describe in detail cardiovascular function in master runners, taking into account the influence of variables such as sex, age, BMI, or physical fitness.

### 4.1. Cardiac Autonomic Function at Rest

The runners’ training focuses mainly on an improvement in endurance capacity, which can induce a reduction in RHR to below 60 bpm, known as sinus bradycardia [41]. In the current study, RHR was in the 37–88 bpm range, similar to those values shown in previous studies [42] with ranges from 30 to 70 bpm. In addition, 46% of the athletes showing bradycardia as a positive adaptation of endurance training. Pronounced resting bradycardia in male elite runners is associated with high HRV [43]. Likewise, in the current study, large correlations were found between HRV and RHR.

Concerning cardiometabolic risk, in the current study, RHR showed only a moderate positive correlation with BMI. RHR is one of the most reliable indicators of health and fitness whereas BMI is used for measuring body composition [44]. In this regard, these authors indicated that a high intensity aerobic exercise program caused a reduction in RHR and a decrease in BMI [44]. Likewise, a cohort study with 9975 people of the general population, Goorakani et al. [45] observed that RHR shows a positive correlation with BMI. Indeed, high BMI values have a positive association with causes of mortality and coronary heart disease, especially in patients with a high RHR [46]. Finally, compared to the present results, Cataldo et al. [47] observed no correlations between 10-km performance and RHR in master endurance athletes.

### 4.2. Cardiac Autonomic Function during Maximal Exercise

The present study shows 178 bpm as the mean HR-peak in master runners. Kwon et al. [48] found a comparable HR-Peak (172.7 ± 11.3 bpm) in endurance-trained athletes during the Bruce protocol. Most athletes displayed HRr ≥ 89 bpm, which is a healthy value, which suggested that our master runners have a heart that is healthy enough to increase the HR according to the required intensity.

Concerning sex, our findings are in general agreement with previous studies in en-durance runners, which showed that HR-peak values were similar in both sexes [19,49,50]. Regarding age and the theory of an age-related decrease in maximum HR [51], our data showed a moderate negative inverse correlation between age and HR-peak, which was not influenced by sex. Similarly, a previous study found correlation coefficients in the order of 0.40 between age and HR max [52].

### 4.3. Cardiac Autonomic Function at Recovery after Exercise

Metaboreflex stimulation (e.g., blood acidosis) is a likely determinant of parasympathetic reactivation in the short term (0–90 min post-exercise); thus, cardiac autonomic recovery is faster in people with a higher aerobic capacity [53]. Moreover, there is a relationship between HRR and PA/aerobic fitness, which suggests that HRR may be a better marker of fitness-related differences in autonomic control in athletes [54]. Bentley et al. [55] revealed that endurance athletes showed a faster HRR in comparison to physically active individuals.

Moreover, abnormal HRR after submaximal exercise (≤42 bpm, 2 min later) is associated, among other factors, with high RHR (≥90 beats/min), high resting systolic blood pressure (≥140 mm Hg), high BMI, low PA levels, and impaired chronotropic response [22]. Regarding the data obtained in our study, we observed that 100% of our participants achieved an HRR > 12 bpm at 1 min and >44 bpm at 5 min, which indicates a healthy HRR after maximal exercise [56]. Likewise, most athletes displayed an HRR_1min_ ≥ 25 bpm [57], and an HRR_5min_ > 75, which is a threshold level for risk of death from any cause.

### 4.4. Heart Rate Variability

Following reference values for the adult population [58], the current study showed that the different HRV parameters found in the runners, and analyzed in absolute values, remaining above the reference values in both men and women. In addition, our data are above the values found by [11,19] in master endurance runners both in the time and frequency domain. An example might be RMSSD (ms) = 38.93 ± 20.44 vs. 51.44 ± 25.87 and LF (ms2) = 2091± 2177 vs. 2555 ± 2150.

In the current study, HRV was influenced by the sex of the participants, with women exhibiting lower values of SDNN and LF than men. The findings of the current study support the previous research which reported substantial sex differences in HRV, both in endurance athletes [59] and in the healthy population [39,60]. The autonomic control of the female heart is characterized by the relative dominance of vagal parasympathetic activity, showing lower SDNN, and greater HF and less LF power than men despite the greater mean HR (smaller RR intervals), whereas the male heart is characterized by relative sympathetic dominance, despite lower HR [60]. These sex differences could be due to developmental differences or the effects of prevailing levels of male and female sex hormones [61]. Indeed, the menstrual cycle could be another factor responsible for fluctuations in cardiac vagal activity [62].

Regarding age, in the present study, no association was found between age and HRV. However, the age-dependency of HRV could be due to relevant cardiovascular alterations, both structural, such as the loss of sinoatrial pacemaker cells or arterial dispensability, and functional, such as altered coupling between regulatory components [63]. Furthermore, this decline in parasympathetic control with age may be partly due to a decrease in physical fitness with age [59]. In this sense, endurance run practice could reduce the declination of HRV along with aging. Therefore, higher cardiorespiratory fitness is associated with higher HRV [64,65].

### 4.5. Relationship between the Different HR Behaviors

It is noteworthy that our findings indicated that HRR_5min_ shows a significantly larger correlation with HRr and CI. Regarding this, Jae et al. [66] indicated that these two parameters, HRR and CI, are related and commonly used as markers of autonomic nervous system dysfunction. Therefore, and by previous studies [67,68], our finding noted that master runners displayed healthy values of chronotropic competence and HRR.

Additionally, we found that HRV showed moderate correlations with HRr. This finding is in agreement with Voss et al. [63], who observed that individuals with high chronotropic competence and HVR values displayed healthy cardiovascular status [69]. In addition, another finding of our study was that HRV exhibits a significantly large inverse correlation with RHR. Similarly, recent research noted that HRV is strongly associated with RHR in endurance-trained runners, which in turn, is related to heart size, which could be because exercise causes morphological and electrical remodeling of the heart, resulting in a lower HR and greater HRV [70].

Overall, following Freeman et al. [52], there is a need to underline that HRr or HRR and several components of HRV, both at rest and during exercise, could describe the complex interaction between the autonomic nervous system and cardiovascular system during exercise testing and could provide some prognostic values of the health status of people, especially, from athletes. Likewise, RHR, HRr, HR-peak, HRR, and HRV can be used to analyze several training adaptations and to assess the effects of autonomic function on the cardiovascular response to exercise [52,71].

Humans are gradually moving toward an epoch where cardiovascular health re-quires looking at the main variables that impact on life span [72]. Numerous studies in the literature suggest that resting HR is contrariwise associated with life span among homeothermic mammals and within individual species [73]. HR is not simply an indica-tor of cardiovascular system status, it is also an indicator of cardiac autonomic nervous system activity (sympathetic and parasympathetic) and metabolic rate [73]. A recent study has reported that HR variability have a function in exceptional longevity in centenarians [74]. For that reason, HR measures could be related to human health. Therefore, the current study is relevant to characterize cardiac behavior in today’s growing population, showing quite pertinent data.

Several limitations of this study must be highlighted. First, the primary limitation of this study is the use of a cross-sectional design; following runners over time in longitudinal research would provide more accurate data regarding aging influence and training effects on cardiovascular autonomic modulation. Second, a priori analysis of sample size was not calculated, unfortunately, we could not get access to the large number of runners we needed because our inclusion criteria were very strict. Therefore, as no a priori sample size calculation was performed, this study should be considered as a pilot study. In addition, the sample included Caucasian Spanish, so a generalization to the wider population should be performed with caution. Third, the HR response needs to consider the mode of exercise test (i.e., treadmill versus bicycle). Fourth, the menstrual cycle was not controlled in our study. Notwithstanding these limitations, our results offer some insights into cardiovascular autonomic modulation in master runners during typical field tests such as 3-km, reinforcing the ecological validity of this study. Moreover, this study provides useful data for sports physicians, coaches, and athletes.

## 5. Conclusions

Age, sex, fitness, or anthropometrics characteristics did not show a relevant influence on cardiovascular autonomic modulation in master runners. However, the 3-km performance displayed a significant negative association with several factors of cardiometabolic risk. From a practical point of view and considering the lack of reference values for assessing the autonomic cardiac function during rest, maximal exercise, and recovery of master runners, the values obtained in this study might play a key as a pilot study on the cardiac response of master’s athletes to effort, which will provide us with indicative clinical information to continue advancing in this research topic. In addition, these values can be used as a ‘warning signal’, where it would be necessary to conduct additional tests to identify possible cardiac diseases.

## Figures and Tables

**Table 1:** Age, anthropometric characteristic of participants and 3-km performance according to sex.

	All (*n* = 50) Mean (SD)	Men (*n* = 29) Mean (SD)	Women (*n* = 21) Mean (SD)	*p*-Values
Age (years)	43.28 (5.25)	43.86 (5.35)	42.47 (5.26)	0.367
Body mass (kg)	65.33 (9.55)	71.01 (7.29)	57.49 (6.15)	<0.001
Body height (cm)	168.36 (8.25)	173.1(6.85)	161.8 (4.83)	<0.001
BMI (kg/m^2^)	22.66 (2.16)	23.63 (1.95)	21.31 (1.71)	<0.001
Body fat (%)	14.62 (6.96)	9.21 (2.51)	21.84 (3.50)	<0.001
WC (cm)	79.98 (6.79)	83.86 (5.28)	74.61 (4.68)	<0.001
WtHR	0.47 (0.03)	0.48 (0.04)	0.46 (0.02)	0.015
3-km race (s)	732.26 (97.71)	661.89 (44.12)	829.42 (62.93)	<0.001
RPE (6–20)	14.60 (1.46)	14.96 (1.31)	14.10 (1.55)	0.043

SD: standard deviation; BMI: body mass index. WC: waist circumference. WtHR: waist-to-height ratio RPE: rate of perceived exertion.

**Table 2:** Cardiovascular autonomic modulation at rest, during exercise and following maximal exercise in master runners well trained.

	All (*n* = 50) Mean (SD)	Men (*n* = 29) Mean (SD)	Women (*n* = 21) Mean (SD)	*p*-Values
RHR (bpm)	59.50 (12.21)	57.32 (13.06)	62.55 (10.49)	0.127
Average HR exercise (bpm)	169.77 (9.27)	169.60 (9.84)	170.00 (8.69)	0.738
HR-peak (bpm)	178.10 (10.62)	177.78 (11.05)	178.52 (10.26)	0.968
HRr (bmp)	117.68 (15.64)	118.57 (17.47)	116.45 (12.99)	0.594
Chronotropic index	1.01 (0.10)	1.00 (0.12)	1.01 (0.07)	0.908
HRR_1min_ (bpm)	48.29 (10.51)	49.00 (10.13)	47.42 (11.14)	0.319
HRR_5min_ (bpm)	83.31 (8.57)	85.46 (7.61)	80.66 (9.13)	0.097
RMSSD (ms), at rest	51.44 (25.87)	57.59 (29.35)	43.15 (17.76)	0.085
SDNN (ms), at rest	100.25 (35.87)	111.59 (37.45)	84.95 (27.70)	0.012
HF (ms^2^), at rest	4946.74 (3512.39)	5938.70 (4059.21)	3607.59 (2008.59)	0.156
LF (ms^2^), at rest	2555.31 (2150.96)	3056.90 (2451.63)	1878.15 (1461.59)	0.045
LF/HF, at rest	2.63 (1.7)	2.77 (1.91)	2.44 (1.43)	0.747

SD: standard deviation. Bpm: beats per minute. RHR: Resting heart rate. HRr: heart rate reserve. HRR: heart rate recovery. RMSSD: square root of the mean squared differences of successive RR intervals. SDNN: standard deviation of all normal R-R intervals. HF: high frequency band. LF: low frequency band. LF/HF ratio: ratio of LF and HF frequency band powers.

**Table 3:** Partial correlations of heart rate parameters with cardiorespiratory fitness and anthropometric variables controlled by age and sex.

Variables	r	*p*-Values
RHR vs. BMI	0.352	0.016
HR-peak vs. age	−0.369	0.009
HR-peak vs. HRR_5min_	0.476	0.001
HRR_5min_ vs. HRr	0.542	<0.001
HRR_5min_ vs. CI	0.495	0.001
RMSSD vs. RHR	−0.741	<0.001
SDNN vs. RHR	−0.646	<0.001
HF vs. RHR	−0.566	<0.001
LF vs. RHR	−0.447	0.002
LF/HF vs. RHR	0.486	0.001
HRr vs. RMSSD	0.505	0.001
HRr vs. SDNN	0.484	0.001
HRr vs. HF	0.374	0.015
HRr vs. LF	0.455	0.002
3 km time vs. BMI	0.314	0.028
3 km time vs. WtHR	0.288	0.045
3 km time vs. body fat	0.374	0.010

BMI: body mass index; RHR: Resting heart rate. WtHR: waist-to-height ratio. HRR: heart rate recovery. RMSSD: square root of the mean squared differences of successive RR intervals. SDNN: standard deviation of all normal R-R intervals. HF: high frequency band. LF: low frequency band. LF/HF ratio: ratio of LF and HF frequency band powers.
